# Acetabular reconstruction for primary and revision total hip arthroplasty using Kerboull-type acetabular reinforcement devices—case–control study with factors related to poor outcomes of surgery

**DOI:** 10.1097/MD.0000000000016090

**Published:** 2019-07-05

**Authors:** Yoshinobu Masumoto, Shigeo Fukunishi, Tomokazu Fukui, Yu Takeda, Shoji Nishio, Yuki Fujihara, Shohei Okahisa, Taishi Okada, Shinichi Yoshiya

**Affiliations:** Department of Orthopedic Surgery, Hyogo College of Medicine, Mukogawa-cho, Nishinomiya City, Hyogo, Japan.

**Keywords:** acetabular bone defect, bone graft, Kerboull-type acetabular reinforcement device, risk factor, total hip arthroplasty

## Abstract

Kerboull-type acetabular support rings (KT) and allogenic bone graft were used for severe periacetabular bone loss with primary and revision total hip arthroplasty (THA). The purpose of this case–control study is to evaluate the risk factors related to poor outcomes of surgery.

Sixty patients underwent primary THA and revision THA using allogenic bone graft with KT for large acetabular deficiency. These patients were retrospectively evaluated postoperatively and followed-up by radiograph. The minimum follow-up period was 4 years and averaged 7 years. A radiological failure was defined by the following criteria:

1) substantial migration defined as change in the angle of inclination of more than 5 degrees or migration of more than 5 mm,
2) breakage of the screw or device,
3) acetabular revision due to aseptic loosening.

Expected risk factors were defined as female, age >75 years, body mass index (BMI) >25%, medical history of hypertension, renal failure, liver steatosis, diabetes, hyperlipidemia, cardiac infarction, smoking, American Academy of Orthopedic Surgery (AAOS) classification III or IV, bleeding>500 mL, time of surgery >3hours, high hip center-type KT, inclination of KT >45 degrees, screw angle >25 degrees, morselized bone graft, Kawanabe's classification stage 3 or 4 and revision surgery. Nineteen hips (31.6%) revealed radiological failure according to our criteria, and revision THA was performed in 2 hips (3.3%). In the statistical analysis, morselized bone graft and high hip center-type KT were identified as factors of poor outcomes of surgery.

## 1 Introduction

Management of periacetabular bone loss during total hip arthroplasty (THA) and revision THA is an ongoing challenge for orthopedic surgeons. Garbuz proposed that the goals of the revision THA on the acetabular side are to provide support for the cup to reapproximate the normal anatomy and to restore the length of the lower limb.^[1]^ A bone graft can be used to accomplish these goals. In addition, these bone grafts restore bone stock, which is a benefit should a future repeat revision become necessary. Multiple treatment options have been described for the reconstruction of acetabular bone deficiencies, including the use of autograft or allograft bone in conjunction with cemented or cementless components. Harris advocated replacement of the acetabular component at a high hip center at the time of revision.^[2]^ High placement of the cup makes it possible to avoid the use of a structural graft; however, the risk of impingement and dislocation increases. Placement of the cup at the correct anatomical level appears to decrease the risk of impingement and dislocation. A bone graft is necessary to place the cup at the original center of rotation. However, several authors described poor results in revision THA with a structural bone graft without a reinforcement device.^[3–7]^ Gerber and Mulroy initially published good results using a structural bone graft for acetabular reconstruction at 7.1 years of follow-up; however, the number of revisions approximately doubled after an additional 5 years.^[8,9]^ Garbuz described that a large structural allograft that supports more than 50% of the cup is necessary to reconstruct the acetabulum at the correct anatomical level for hips with severe deficiency of the acetabular bone stock.^[1]^ When the graft supports more than 50% of the cup, they advocate the use of a reconstruction device and a cup inserted with cement. On the other hand, major available devices have been used for acetabular revision, including Muller support ring, Burch-Schneider anti-protrusion cage, Ganz plate and Kerboull acetabular reinforcement device (Kerbull device). The Kerboull device was developed in 1974 and the Kerboull-type plate (Kyocera Medical, Osaka, Japan) is a similarly shaped, modified Kerboull device that KT introduced in 1993.^[10,11]^ Additionally, KT provides variations in offset length and vertical length.

In the present study, 60 hips that underwent THA or revision THA with severe acetabular bone defect and underwent acetabular reconstruction using allogenic bone graft with KT between 1995 and 2014 were retrospectively evaluated. The focus was on factors related to poor outcomes of surgery.

## 2 Materials and methods

This study was approved by the Institutional Review Board of the Hyogo College of Medicine (No. 2190), and informed consent was obtained from all patients.

### 2.1 Study population

Seventy patients underwent primary THA and revision THA using allogenic bone graft with KT for large acetabular deficiency between 1995 and 2014. Among these 70 patients, 2 patients underwent revision surgery due to deep infection and 8 patients were lost during the study period without a follow-up. These 10 patients were excluded from this study. In total, 60 patients were included in this retrospective study. There were 10 male and 50 female patients with a mean age of 65.9 years (range, 25–86 years). The minimum follow-up period was 4 years and averaged 7 years (range, 4–16 years). During the follow-up periods, 11 patients died for reasons unrelated to THA, and the final follow-up period for these patients was defined as the time of their death. There were 25 primary THAs and 35 revision THAs. Hip pathologies in primary THA included osteoarthritis in 2 hips, rapidly destructive coxarthropathy in 3 hips, rheumatoid arthritis with protrusio acetabuli in 12 hips, post-traumatic osteoarthritis in 4 hips, hemophilic arthritis in 1 hip, and hemodialysis-related arthritis in 3 hips. During surgery, the acetabular defect was classified according to the American Academy of Orthopedic Surgery (AAOS) system of grading and the total number of each type is as follows: Type I in 19 hips, Type II in 15 hips, Type III in 23 hips, and Type IV in 3 hips. Additional grading by Kawanabe's classification of the acetabular bone defect was performed according to the severity of the superior segmental bone loss.^[12]^ Stage 1 indicates no superior bone loss while the whole pallet of the KT is placed against the host bone. Stage 2 indicates superior bone loss, with more than 50% of the pallet in contact with the host bone. Stage 3 indicates superior bone loss with less than 50% of the palette touching the host bone. Stage 4 indicates massive bone loss with no part of the pallet touching the host bone. There were 14 hips with stage 1, 12 hips with stage 2, 13 hips with stage 3, and 21 hips with stage 4.

### 2.2 Surgical procedure

**Figure 1:**
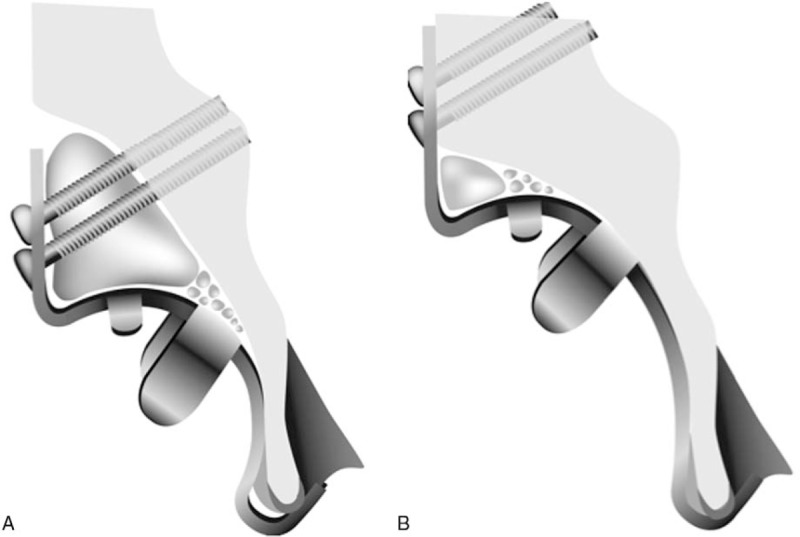
Schematic drawing of the 2 types of KT and bone graft. A. OC-type KT. OC-type KT could place the cup in origizal acetabulum. B. HH-type KT. The HH-type KT could decrease the volume of bone graft and enables the cup to be placed in the high hip center. HH = high hip center-type, KT = Kerbull-type acetabular support rings, OC = original cup center-type.

All procedures were performed by 1 senior surgeon (SF) who is experienced in the surgical technique for revision THA. In the THA procedure, surgeries were performed in the lateral position with a direct-lateral approach. Allogenic bone graft was used for all included patients. The graft bone was obtained from the femoral head of osteoarthritis patients during the previous primary THA, and it was harvested under sterile conditions using a Lobator sd-2 (Telos, Marburg, Germany) and stored at −80 degrees. These procedures were performed according to the guidelines of the Japanese Orthopedic Society. The morselized bone graft was used for acetabular bone defects in 30 hips until 2007. Since 2007, a structural bone graft was used in 30 hips for superior acetabular bone defects. Additionally, the morselized bone was packed into the gaps between the structural bone graft and host bone. KT was used for all cases as a reinforcement device, and to protect the graft bone. KT has various types of offsets and vertical lengths including a vertical offset of 0 mm, 10 mm, and 15 mm; however, they have been classified into the following 2 types: original cup center-type with a vertical offset of 0 mm (original cup center-type [OC]) and high hip center-type with a vertical offset of 10 mm and 15 mm (high hip center-type [HH]) (Fig. 1 A and B). The HH-type KT could decrease the volume of bone graft while enabling the cup to be placed in the high hip center. An OC-type KT was used in 27 hips and HH-type KT was used in 33 hips. Placement of the KT was performed following the technique described by Kerboull.^[10]^ The hook of the KT plate was placed under the teardrop area and at least 2 screws were passed through the palette of the KT to fix it to the pelvis. In severe cases of AAOS type III or Kawanabe's stage 4, the palette of the KT could not be attached directly to the pelvis due to superior bone defect. In these cases, all screws were fixed to the pelvis through a structural bone graft. Post-operative management included partial weight bearing immediately after surgery, and full weight bearing was allowed 3 to 4 weeks after surgery in all patients.

### 2.3 Postoperative evaluation

Post-operative and follow-up radiographs were compared to assess migration of the implant using the following parameter.

1. Change in the inclination angle of KT and
2. central migration: defined as the distance between the medial wall line and the center of the femoral head. Substantial migration was defined as change in the angle of inclination of more than 5 degrees or migration of more than 5 mm. In addition, the average angle was measured between the inter-teardrop line and direction of screws postoperatively.

Radiological failure was defined by any of the following criteria:

1) substantial migration,
2) breakage or loosening of the screw or device, and
3) acetabular revision due to aseptic loosening.

The focus was on factors related to poor outcomes of surgery, such as radiological failure. Regarding patient-related factors, we evaluated sex, age, body mass index (BMI), medical history of hypertension, renal failure, liver steatosis, diabetes, hyperlipidemia, cardiac infarction, smoking, and AAOS classification before surgery. For surgery-related factors, bleeding, time of surgery, type of KT (OC-type or HH-type), inclination angle of KT, the average angle of the screws, type of bone graft (structural bone or morselized bone), Kawanabe's classification, and the type of surgery (revision THA or primary THA) were evaluated. Expected risk factors were defined as female, age >75 years, BMI >25%, medical history of hypertension, renal failure, liver steatosis, diabetes, hyperlipidemia, cardiac infarction, smoker, AAOS classification III or IV, bleeding >500 mL, time of surgery >3hours, HH-type KT, inclination of KT >45 degrees, screw angle >25 degrees, morselized bone graft, Kawanabe's classification stage 3 or 4, and revision surgery.

### 2.4 Statistical analysis

In the statistical analysis, a univariate analysis of potential risk factors was initially performed using Fisher exact test. Factors that were found to have values of *P* <.05 in the univariate analysis were further analyzed in the multivariate logistic regression analysis. Results were summarized as odds ratios, 95% confidence intervals, and *P* values *P* <.05, which were considered statistically significant. The statistical analysis was performed using SPSS software (ver.19.0, SPSS Inc., Chicago IL).

## 3 Results

**Figure 2:**
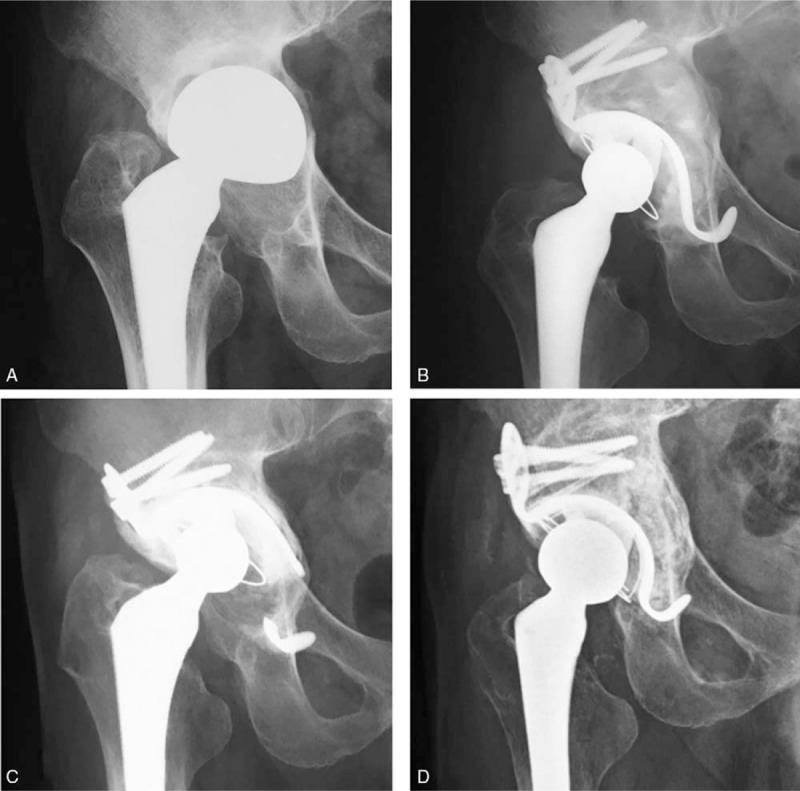
X-ray radiograph of the hip with 57-year-old man. A. at initial visit. B. after first revision surgery with HH-type KT and a structural bone graft. C. ten years after revision surgery. D. after re-revision surgery with OC-type KT and a structural bone graft. HH = high hip center-type, KT = Kerbull-type acetabular support rings, OC = original cup center-type.

**Figure 3:**
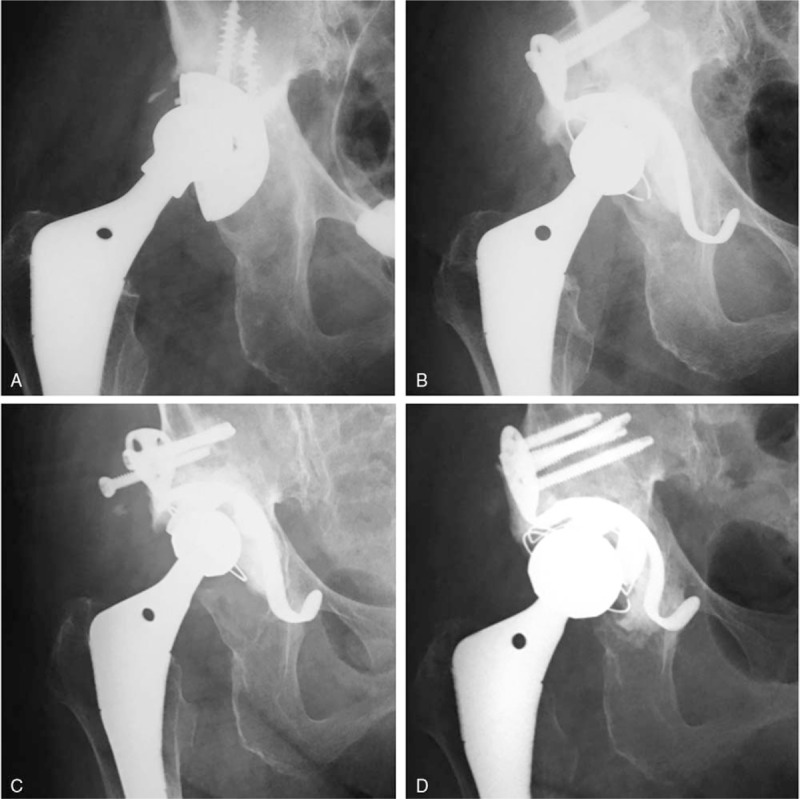
X-ray radiograph of the hip with 77-year-old woman. A. at initial visit. B. after first revision surgery with HH-type KT and a morselized bone graft. C. nine years after revision surgery. D. after re-revision surgery with OC-type KT and a structural bone graft. HH = high hip center-type, KT = Kerbull-type acetabular support rings, OC = original cup center-type.

Nineteen hips (31.6%) revealed radiological failure according to our criteria. Seventeen hips developed substantial migration of KT. Among these 17 hips, 8 hips showed change in the angle of inclination of more than 5 degrees, 13 hips showed migration of more than 5 mm, and 4 hips showed both. Breakage of KT developed in 5 hips, and breakage or loosening of the screws developed in 2 hips. Finally, revision THA was performed in 2 hips (3.3%). The first case was a 57-year-old man who underwent revision THA due to upper migration of bipolar arthroplasty. The acetabular defect was classified as AAOS type III and Kawanabe's grading stage 3 during surgery. Revision THA was performed using a structural bone graft and HH-type KT. Ten years after revision surgery, the patient underwent re-revision THA for the acetabular side due to substantial migration and breakage of the hook in the KT. Re-revision THA was performed using a structural bone graft and OC-type KT (Fig. 2 A–D. The second case was a 77-year-old woman who underwent revision THA due to the loosening of the acetabular component. The acetabular defect was classified as AAOS type I and Kawanabe's grading stage 3 during surgery. Revision THA was performed using a morselized bone graft and HH-type KT. Nine years after revision surgery, the patient underwent re-revision THA for the acetabular side due to substantial migration and the loosening of the screws. Re-revision THA was performed using a structural bone graft and OC-type KT (Fig. 3 A–D). Four cases with breakage of the KT were conservatively managed. Three hips had stable acetabular components and KT instead of breakage of the hook, and the patients had no clinical symptoms. One hip is planned to undergo revision surgery soon due to upper migration of the acetabular component and KT.

In the analysis of risk factors, potential risk factors of radiological failures selected in the univariate analysis with *P* values of less than .05 were demonstrated in HH-type KT, inclination of KT >45 degrees and morselized bone graft. Other examined factors (sex, age, BMI, medical history of hypertension, renal failure, liver steatosis, diabetes, hyperlipidemia, cardiac infarction, smoker, AAOS classification, bleeding, time of surgery, screw angle, Kawanabe's classification, and revision surgery) did not significantly correlate with radiological failure (Table 1). In the subsequent multivariate logistic regression analysis, HH-type KT (*P* = .01, odds ratio 6.8) and morselized bone graft (*P* = .03, odds ratio 4.3) were found to be risk factors for postoperative radiographic failure (Table 2).

**Table 1:**
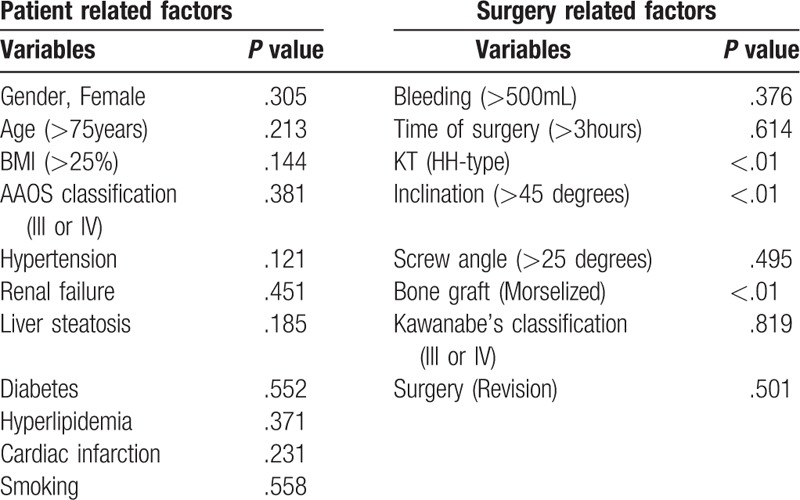
Univariate analysis of risk factors for postoperative radiological failure using Fisher exact test.

**Table 2:**
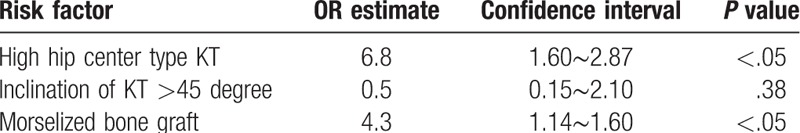
Multivariate logistic regression analysisof risk factors for postoperative radiological failure.

## 4 Discussion

The Kerboull acetabular reinforcement device, which was developed in 1974, is characterized by the fixation of the primary acetabulum with a stainless-steel cup.^[10]^ The device has shown to provide several advantages by protecting bone grafts from over stress, working as a guide for hip reconstruction with the appropriate center of rotation, and stabilizing the reconstructed acetabulum. KT is a similarly shaped, modified Kerboull device introduced in 1993, which consists of a pallet, dome and a hook where screws enter the acetabular bone from outside of the pallet. The device provides a limited variation in the offset length and vertical length. Kerboull described an original technique for AAOS grade III and IV acetabular bone defect reconstruction using a Kerboull device associated with structural allograft for segmental defect reconstruction and morselized allograft for filling a cavity defect.^[10]^ They reported excellent results and mentioned that the survival rate of hips reconstructed using this device along with a bone graft was 92.1% at 13 years. Additionally, several other papers have reported favorable clinical outcomes of using KT and allogenic bone grafting.^[11,13,14]^ However, there have been several reports that have shown a high failure rate of morselized bone graft with acetabular reinforcement devices, including the Kerboull device and KT for acetabular revision surgery.^[12,15–17]^ Morselized bone grafts are superior in terms of remodeling from the host bone compared to structural bone grafts; however, morselized grafts have lower mechanical strength than structural grafts, and breakage of the acetabular reinforcement device may appear before remodeling of the graft bone.^[18–20]^ In regards to the Kerboull device and KT, Kawanabe reported the midterm results of revision THA using a bone graft and a Kerboull device. The survival rate of morselized graft and structural graft at 10 years was 53% and 82%, respectively. He concluded that mid-term results of revision THA with Kerboull devices were better when structural grafts were used for bone defect of any size.^[12]^ Akiyama demonstrated that morselized bone was acceptable as a bone graft material for bone defect measuring less than 25 mm in height.^[16]^ However, Okano reported that 7 of 32 revisions with allograft thickness of more than 20 mm with KT were classified as a failure after a mean follow-up of 6.3 years.^[17]^ Hayashi also reported the survival rate using different types of bone grafts (HA, beta-TCP, and bulk allograft) for revision THA with KT.^[21]^ The survival rate with a mean follow-up duration of 7.4 years was 74.2%, 81.5%, and 94.7% for HA, beta-TCP, and bulk allograft, respectively. In the present study, radiological failure was revealed in 19 hips (19/60, 31.6%), which included 2 revisions (2/60, 3.3%) with a mean follow-up of 7 years. Similarly, radiological failure occurred in 15 hips using morselized bone graft (15/30, 50.0%), and in 4 hips with structural bone graft (4/30, 13.3%). The morselized bone grafts could be a related factor for poor outcomes in acetabular reconstruction using KT.

Another factor for poor outcomes of surgery was revealed through the use of an HH-type KT. There have been no previous papers mentioning this type of KT. In the present study, radiological failure was revealed in 16 hips (16/33, 48.5%) using HH-type KT and in 3 hips (3/27, 11.1%) using OC-type KT. There were significant differences between the 2 types of devices. In addition, 4 of 33 cases (12.1%) revealed breakage of the hook in HH type KT. Kawanabe described the finite element model (FEM) study in which high-stress areas were localized on the hook and the bend of the pallet, but not on the inner side of the device.^[22]^ The HH-type KT has a longer lever-arm between the dome and the hook, and it is hypothesized that over-stress in the hook could continue during the remodeling process of the graft bone. On the other hand, HH-type KT had the advantage when placing the cup at the high hip center, as it made it possible to decrease the volume of bone graft in cases with severe superior bone defect. However, these cases tend to increase the inclination angle of KT in order to attach the pallet to the pelvis. Kamada described that an inclination angle of KT of more than 45 degrees could lead to poor survivorship compared to an inclination angle of less than 45 degrees.^[23]^ Similar results showed that an inclination angle of KT of more than 45 degrees could be identified as one of the factors for poor outcomes of surgery in the univariate analysis in the present study. Several factors could have contributed to the results. When the OC-type KT was selected for cases with severe superior bone defect, a massive bone graft was necessary for the reconstruction. Jasty described that revascularization and osseous ingrowth in a large bulk bone graft after revision surgery takes several years, and may contribute to graft collapse.^[2]^ The graft bone should be partially unloaded and protected from excessive mechanical force during the remodeling process. Recently, Makita described that consolidation of the bone graft was completed within 12 months in Paprosky type 3A and 3B acetabular bone defect,^[24]^ and Oe described that The success rate of bone remodeling as radiological demarcation was 100% at bone-bone interface after acetabular reconstruction with KT in primary THA and 94% in revision THA.^[25]^ In later cases in the present study, we used one whole femoral head allograft for superior bone defects of the acetabulum in the cases of Kawanabe's Type IV. The palette of the KT could not be attached directly to the pelvis in these cases. The pallet was fixed to the pelvis through a femoral head allograft in OC-type KT. However, the whole femoral head allograft with OC-type KT for Kawanabe's Type IV acetabulum did not reveal radiological failure during the follow-up periods. It is hypothesized that the use of the OC-type KT protects the massive structural grafted bone through its screw fixation to the pelvis, thereby partially protecting the grafted bone from excessive loading during the incorporation and remodeling process. On the other hands, Hayashi reported a risk factor for failure of revision THA using KT and proposed that severe acetabular defects, which are AAOS type IV, was a risk factor for radiographic failure.^[21]^ In the present study, however, AAOS classification III and IV did not significantly correlate with radiological failure. Therefore, use of the OC-type KT and structural bone graft were preferred for any type of acetabula defect according to the clinical and radiological results. Following the present study, sufficient structural bone graft seems to be necessary to avoid the inclination of OC-type KT of more than 45 degrees in our current practice in order to improve long term survivorship of the surgery.

*Limitations*: There are several limitations in this study. First, the follow-up period was relatively short and a longer follow-up might have been necessary. Second, using a morselized bone graft resulted as a risk factor, leading to poor outcomes of surgery; however, this has been utilized in our initial series of surgeries between 1995 and 2007, and from 2007 onwards structural bone graft has been used. It is possible that a learning curve exists for improving our surgical technique. Third, the present study retrospectively evaluated radiographs and medical charts. It is not clear why HH-type KT was chosen for surgery of acetabular defects; however, it is possible that HH-type KT was chosen instead of OC-type KT for cases of severe acetabular deficiency.

*Future directions:* Current surgical options for severe acetabular deficiency in THA or THA revision is will follow the results of this retrospective study. We used OC-type KT for the cup placed original acetabulum and sufficient volume of the structural bone graft was used in order to avoid an inclination angle of KT of more than 45 degrees.

In conclusion, the morselized bone graft and HH-type KT resulted in becoming risk factors for primary and revision THA with acetabular reconstruction using allogenic bone graft and KT in severe acetabular deficiency.

## Acknowledgment

The authors thank Mr Devin Casadey and Ms Rebecca Imaizumi for their assistance in editing the English of this manuscript.

## Author contributions

**Conceptualization:** Shigeo Fukunishi.

**Data curation:** Yoshinobu Masumoto, Tomokazu Fukui, Yu Takeda, Shoji Nishio, Yuki Fujihara, Shohei Okahisa, Taishi Okada.

**Formal analysis:** Yoshinobu Masumoto, Tomokazu Fukui, Yu Takeda, Shoji Nishio, Yuki Fujihara, Shohei Okahisa, Taishi Okada.

**Funding acquisition:** Yoshinobu Masumoto, Tomokazu Fukui, Yu Takeda, Shoji Nishio, Yuki Fujihara, Shohei Okahisa, Taishi Okada.

**Investigation:** Yoshinobu Masumoto, Tomokazu Fukui, Yu Takeda, Shoji Nishio, Yuki Fujihara, Shohei Okahisa, Taishi Okada.

**Methodology:** Shigeo Fukunishi, Yu Takeda.

**Project administration:** Shigeo Fukunishi.

**Resources:** Shigeo Fukunishi.

**Supervision:** Shigeo Fukunishi, Shinichi Yoshiya.

**Validation:** Shigeo Fukunishi, Shinichi Yoshiya.

**Visualization:** Yoshinobu Masumoto.

**Writing – original draft:** Yoshinobu Masumoto.

**Writing – review & editing:** Shigeo Fukunishi, Shinichi Yoshiya.
